# Repeated batches as a strategy for high 2G ethanol production from undetoxified hemicellulose hydrolysate using immobilized cells of recombinant *Saccharomyces cerevisiae* in a fixed-bed reactor

**DOI:** 10.1186/s13068-020-01722-y

**Published:** 2020-05-11

**Authors:** Thais S. Milessi, Caroline L. Perez, Teresa C. Zangirolami, Felipe A. S. Corradini, Juliana P. Sandri, Maria R. Foulquié-Moreno, Roberto C. Giordano, Johan M. Thevelein, Raquel L. C. Giordano

**Affiliations:** 1 Department of Chemical Engineering, Federal University of São Carlos, Rodovia Washington Luís, km 235, 13565-905 São Carlos, SP Brazil; 2 Institute of Natural Resources, Federal University of Itajubá, Av. Benedito Pereira dos Santos, 1303, 37500-903 Itajubá, MG Brazil; 3 Graduate Program of Chemical Engineering, Federal University of São Carlos (PPGEQ-UFSCar), Rodovia Washington Luís, km 235, 13565-905 São Carlos, SP Brazil; 4 Laboratory of Molecular Cell Biology, Institute of Botany and Microbiology, KU Leuven, Kasteelpark Arenberg 31, 3001 Leuven-Heverlee, Flanders Belgium; 5 Center for Microbiology, VIB, Kasteelpark Arenberg 31, 3001 Leuven-Heverlee, Flanders Belgium

**Keywords:** Hemicellulose utilization, Cell immobilization, Inhibitor tolerance, Bioethanol, Recombinant yeast, Fixed-bed reactor, Lignocellulosic biomass

## Abstract

**Background:**

The search for sustainable energy sources has become a worldwide issue, making the development of efficient biofuel production processes a priority. Immobilization of second-generation (2G) xylose-fermenting *Saccharomyces cerevisiae* strains is a promising approach to achieve economic viability of 2G bioethanol production from undetoxified hydrolysates through operation at high cell load and mitigation of inhibitor toxicity. In addition, the use of a fixed-bed reactor can contribute to establish an efficient process because of its distinct advantages, such as high conversion rate per weight of biocatalyst and reuse of biocatalyst.

**Results:**

This work assessed the influence of alginate entrapment on the tolerance of recombinant *S. cerevisiae* to acetic acid. Encapsulated GSE16-T18SI.1 (T18) yeast showed an outstanding performance in repeated batch fermentations with cell recycling in YPX medium supplemented with 8 g/L acetic acid (pH 5.2), achieving 10 cycles without significant loss of productivity. In the fixed-bed bioreactor, a high xylose fermentation rate with ethanol yield and productivity values of 0.38 g_ethanol_/g_sugars_ and 5.7 g/L/h, respectively were achieved in fermentations using undetoxified sugarcane bagasse hemicellulose hydrolysate, with and without medium recirculation.

**Conclusions:**

The performance of recombinant strains developed for 2G ethanol production can be boosted strongly by cell immobilization in alginate gels. Yeast encapsulation allows conducting fermentations in repeated batch mode in fixed-bed bioreactors with high xylose assimilation rate and high ethanol productivity using undetoxified hemicellulose hydrolysate.

## Background

In the last decade, second-generation bioethanol production has received worldwide attention due to the search for sustainable energy sources and the reduction of greenhouse gas emissions. Lignocellulosic feedstocks have great potential as raw materials for 2G ethanol production since they are cheap and rich in fermentable sugars (mainly glucose and xylose), which can be obtained from their cellulose and hemicellulose content [1]. In addition, these raw materials generally do not compete, direct or indirectly, with food/feed demands [2].

In order to make 2G ethanol production from lignocellulosic biomass economically feasible, all fermentable sugars present in the lignocellulosic feedstock must be optimally utilized [3]. The production of ethanol from hexoses with *Saccharomyces cerevisiae* is well established at industrial scale using sucrose as carbon source. This yeast has been generally employed for ethanol production due to its superior ethanol yield and tolerance to high ethanol and sugar concentrations [4], being a good choice to produce ethanol from glucose, the main component of cellulose fraction of lignocellulosic biomass. However, as *S. cerevisiae* cannot metabolize xylose and since other species of microorganisms that are naturally capable of metabolizing xylose lack the advantages of *S. cerevisiae* for industrial employment [3, 4], the hemicellulose fraction is underutilized, making the development of microorganisms capable of assimilating the xylose from this fraction a crucial research goal.

The application of metabolic engineering for improvement of *S. cerevisiae* xylose assimilation has been intensively studied due to its promising potential [4–7]. Among the strategies used, insertion of genes for either xylose reductase and xylitol dehydrogenase (XR + XDH) or xylose isomerase (XI) have been the two major options. However, the expression of the cofactor-independent heterologous XI pathway seems to be the best approach due to the redox balance problem with the XR/XDH pathway under microaerobic and anaerobic conditions [8].

In spite of the breakthroughs in identifying XI enzymes with high activity upon expression in yeast, there are still many challenges in developing an efficient and robust strain, displaying high fermentation rates and low byproduct formation in concentrated, undetoxified lignocellulose hydrolysates [1, 9].

In addition to constructing efficient recombinant *S. cerevisiae* strains, the development of competitive and robust processes for 2G ethanol production also depends on optimizing the technology for long-term bioreactor operation with a high load of viable yeast cells, using concentrated lignocellulose hydrolysate as fermentation medium. While these conditions are crucial to attain high ethanol productivity and titers, they intensify the exposure of yeast cells to toxic compounds (including ethanol itself) present in the fermentation broth, thus compromising the fermentation performance of *S. cerevisiae* [10].

The use of lignocellulosic materials as feedstock requires a pretreatment step to promote disruption of the fibrous matrix and release of the fermentable sugars. However, during this process inhibitory compounds are generated, such as acetic acid, hydroxymethylfurfural and furfural [7].

The presence of acetic acid in lignocellulose hydrolysates is unavoidable, since it originates from the hemicellulose xylan acetyl side groups. This compound significantly reduces yeast fermentation ability even at low concentrations [11]. The artificial capacity to ferment pentoses accomplished by genetic engineering is especially sensitive to acetic acid [7, 12]. Thus, strategies to overcome the low tolerance of yeast to this compound must be developed in order to achieve efficient and total conversion to ethanol of the sugars present in the hydrolysate [12]. In most published studies, the exposure to inhibitors is addressed by improving the tolerance of the yeast itself using approaches based on evolutionary and/or metabolic engineering to obtain more tolerant phenotypes [4, 10]. In this work, cell immobilization by gel entrapment, a known technique to enable process operation at high cell loads and productivities [13], is revisited and evaluated as an alternative or additional strategy to handle inhibition.

Cell immobilization has several advantages, e.g., the easy recovery of the biocatalyst for recycling, the possibility of operating the bioreactor with high biocatalyst densities. Besides, immobilization may build up a microenvironment inside the beads that may help in protecting the cells against external harmful factors [13]. Yeast cell immobilization in different solid matrices has been employed frequently for 1G ethanol production [14, 15]. On the other hand, there are only few reports on 2G ethanol production using *S. cerevisiae* entrapped cells [16, 17]. In the case of GMO yeasts, the use of immobilized cells is particularly suitable for several reasons. First of all, it strongly reduces the effort to produce, transport and store enough biomass of the recombinant yeast. In addition, it helps to isolate the recombinant strain from any contaminants and to cope with legal restrictions for use of GMOs in some countries, such as Brazil [18, 19].

Furthermore, the use of a fixed-bed reactor can also contribute to an economically feasible process because of its specific advantages, such as high conversion rate per weight of biocatalyst, high productivity and reduced cost of operation and maintenance, due to the possibility of long operating periods and easy biocatalyst recovery [20, 21]. The fixed-bed bioreactor allows process operation in repeated batches, which is an interesting approach for bioethanol production using immobilized yeast cells since the microorganism can be immediately reutilized in a new fermentation batch, with no need of a centrifugation step, reducing process time and cost and consequently improving productivity [22].

In this context, the present work evaluated 2G ethanol production from undetoxified sugarcane bagasse hemicellulose hydrolysate by recombinant *S. cerevisiae* in a fixed-bed bioreactor operating in repeated batches. The hemicellulose hydrolysate was obtained after an acid hydrolysis pretreatment of sugarcane bagasse, representing a worst-case scenario with respect to the presence of inhibitors such as furfural and hydroxymethylfurfural (HMF) [23, 24].

First, to evaluate the benefits of the immobilization technique for protection against inhibitor toxicity, the performance of free and immobilized yeast was compared in a repeated batch process using xylose-rich medium with different concentrations of acetic acid. Due to the excellent performance of the yeast, operation of the fixed-bed reactor system with non-detoxified sugarcane bagasse hemicellulose hydrolysate resulted in remarkably high ethanol yields and productivities considering the high inhibitor content of the fermentation medium.

## Results

### Xylose fermentation by T18 yeast in the presence of acetic acid

**Fig. 1:**
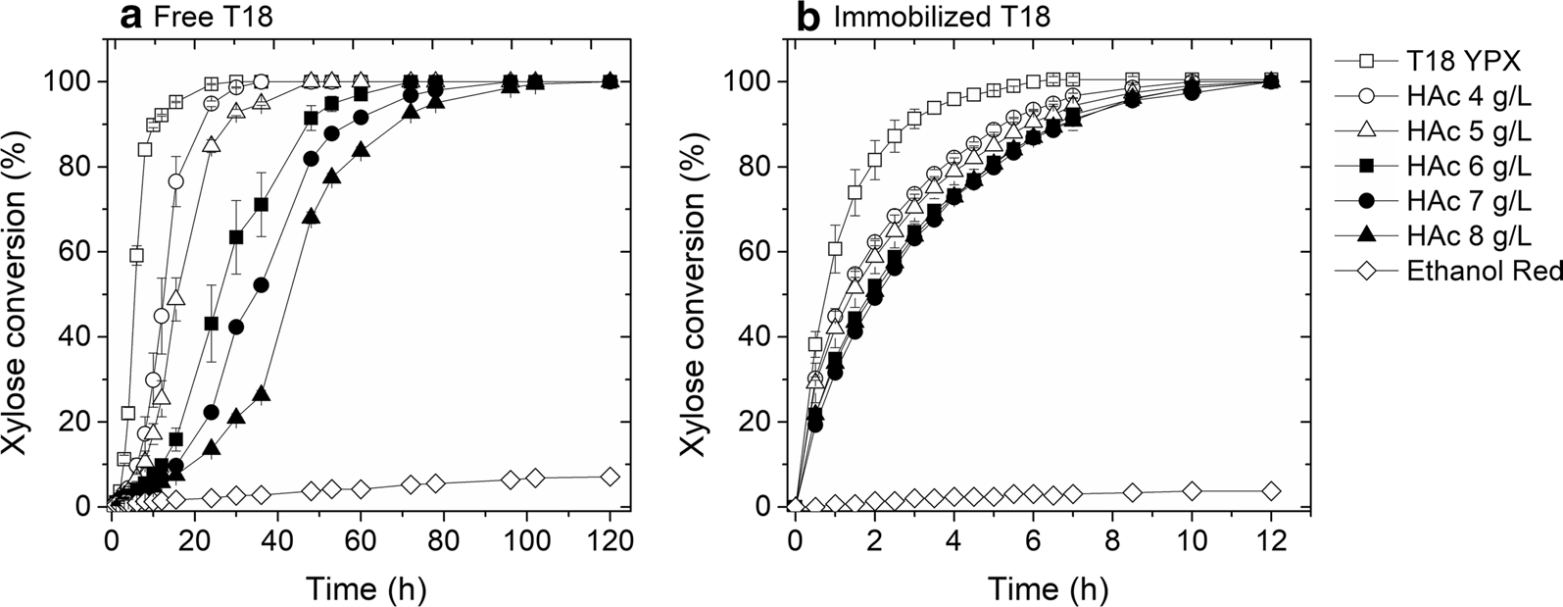
Profiles of YPX (40 g/L) fermentation by T18 in the presence of different acetic acid concentrations. **a** Free cells (OD_0_ = 4; 2 g_drycells_/L) and **b** immobilized cells (1:1 *V*_beads_/*V*_medium_, OD_0_ = 100; 50 g_drycells_/L) (35 °C, 150 rpm and pH 5.2). The Ethanol Red strain was used as negative control

The performance of the recombinant *S. cerevisiae* GSE16-T18 (T18) strain in free and immobilized form was evaluated for xylose fermentation in the presence of different concentrations of acetic acid (0 to 8.0 g/L). Free cells of T18 (OD_0_ = 4; 2 g_drycells_/L) consumed xylose efficiently (40 g/L in 12 h) (Fig. 1a), with an ethanol productivity of 1.36 g/L/h and yield of 0.441 g_ethanol_/g_xylose_ (Additional file 1). No significant formation of byproducts, such as xylitol, was detected.

The performance of immobilized T18 yeast (OD_0_ = 100; 50 g_drycells_/L), on the other hand, was exceedingly superior than that of free cells, and the deleterious effect of acetic acid was effectively reduced (Fig. 1b). The fermentation without acetic acid lasted only 4 h (with a productivity of 3.6 g/L/h), three times faster than the experiment using free yeast. The entrapment in alginate gel attenuated the influence of acetic acid and enabled the consumption of all xylose in at most 12 h for all tested acetic acid concentrations. Thus, the fermentation using immobilized T18 was one order of magnitude faster than that of free T18 cells. For example, in the presence of 8 g/L of acetic acid, the fermentation time improved from 120 to 12 h.

Part of this improvement in productivity is due to the characteristic of bioreactors working with immobilized cells, i.e., of supporting higher concentrations of the catalyst. This is an advantage from the point of view of the industrial process, but may mask the role of the microenvironment created inside the alginate beads on the fermentation performance. To clarify this question, an additional experiment in YPX 40 g/L containing 8 g/L of acetic acid was carried out using the same initial concentration of free and immobilized cells (50 g_drycells_/L_reactor_) (Table 1). The results confirm that there is a protective effect of the calcium alginate gel. Using the encapsulated yeast, the productivity more than doubled when compared to the free cells, including for assays with the same initial load of cells in the reactor.

**Table 1:** Fermentation performance of free and encapsulated T18 in YPX with acetic acid

	T18-free cells	T18-encapsulated cells
Initial xylose (g/L)	40.1	34.5
Final ethanol (g/L)	15.3	15.8
Residual xylose (g/L)	3.9	0
Y_P/S_ (g/g)	0.423	0.458
Q_P_ (g/L/h)	0.638	1.317
q_p_ (mg/g_drycells_/h)	12.8	26.3
Fermentation time (h)	24	12

Experimental conditions: YPX + 8 g/L acetic acid, initial concentration of 50 g_drycells_/L (OD_0_ = 100), 35 °C, 150 rpm and pH 5.2. Values are triplicates averages, with less than 5% standard error

Due to the excellent performance of immobilized T18 at high concentrations of acetic acid, the maximum concentration of acetic acid at which the immobilized yeast would still be capable of efficiently fermenting xylose was determined. Encapsulated T18 was capable of fermenting xylose efficiently in the presence of concentrations of acetic acid up to 11 g/L (Additional file 2). These results support the potential of yeast immobilization to achieve very high acetic acid tolerance. The immobilization procedure was efficient since the integrity of biocatalyst beads was unchanged at the end of the process. Despite high cell densities inside the beads, there was no observable loss of cells into the medium.

### Repeated batch xylose fermentation by immobilized T18 yeast

**Fig. 2:**
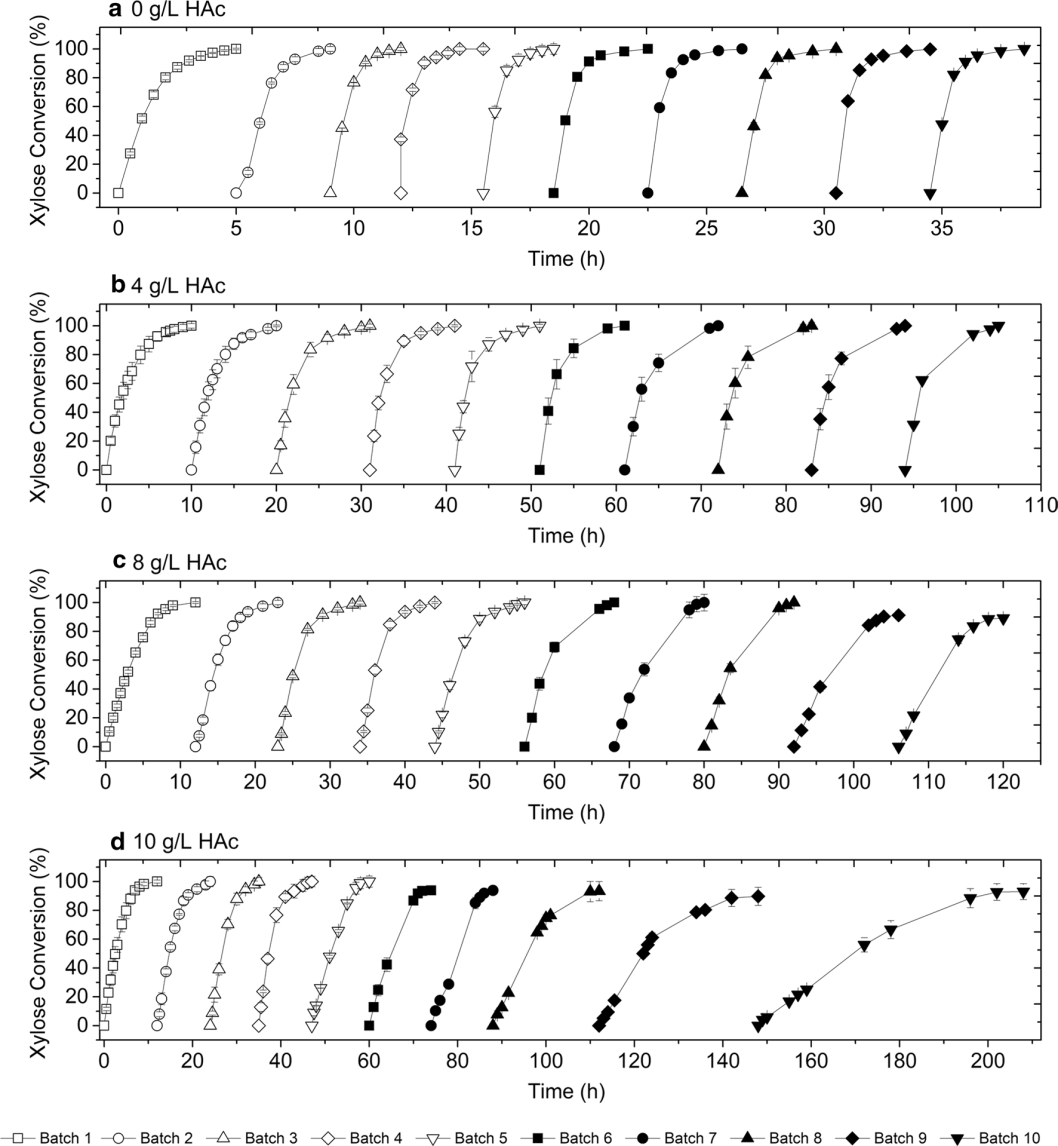
Repeated batch fermentations with encapsulated T18 in YPX 40 g/L with different acetic acid concentrations (DO_0_ = 100; 50 g_drycells_/L, 35 °C, 150 rpm and pH 5.2)

Repeated batch xylose fermentation experiments were performed in the presence of different concentrations of acetic acid. The experiments were carried out as repeated batches with 50 g_drycell_/L of immobilized cells, and they were ended after 10 cycles because at that moment more than 10% of the initial bead mass had been withdrawn for quantification of cell viability (Fig. 2a–d). Encapsulated T18 was capable of fermenting 10 repeated batches in YPX 40 g/L with the same performance (Fig. 2a), achieving an ethanol titer of 17 g/L and a yield of 0.44 g_ethanol_/g_xylose_, without byproduct formation. Cell viability remained constant in all batches (Table 2).

**Table 2:** Initial ethanol and acetic acid concentrations, ethanol production and cell viability during repeated batches with encapsulated T18

	Batch									
	1	2	3	4	5	6	7	8	9	10
A										
Cell Viability (%)	100	97	98	97	98	98	98	98	97	98
[EtOH]_0_ (g/L)	0	5.0	4.8	6.5	6.6	5.9	5.6	5.5	6.0	5.8
[HAc]_0_ (g/L)	0	0	0	0	0	0	0	0	0	0
[EtOH] (g/L)	16.6	21.4	24.2	21.2	20.9	24.6	20.8	23.3	23.4	24.7
*Q*_P_ (g/L/h)	4.2	4.1	4.7	4.2	4.1	4.7	4.3	4.5	4.4	4.6
B										
Cell viability (%)	99	98	98	98	97	98	98	97	96	92
[EtOH]_0_ (g/L)	0	4.8	6.1	6.9	6.3	7.0	7.1	6.4	7.6	6.7
[HAc]_0_ (g/L)	4.3	7.7	9.6	8.3	10.4	10.3	9.7	10.3	10.7	10.7
[EtOH] (g/L)	16.1	22.7	23.8	25.0	24.6	25.8	25.6	26.8	27.5	27.8
Q_P_ (g/L/h)	1.6	1.8	1.6	1.8	1.8	1.9	1.7	1.9	1.8	1.9
C										
Cell viability (%)	99	98	98	98	98	98	98	84	81	72
[EtOH]_0_ (g/L)	0	4.1	6.0	6.1	5.9	5.8	5.8	5.4	6.0	6.1
[HAc]_0_ (g/L)	7.9	11.7	13.3	13.2	14.0	14.0	14.4	14.2	14.4	14.8
[EtOH] (g/L)	15.8	22.4	23.8	23.7	23.2	22.9	23.3	24.0	24.0	24.6
Q_P_ (g/L/h)	1.6	1.7	1.6	1.8	1.4	1.4	1.5	1.5	1.3	1.6
D										
Cell viability (%)	99	98	98	98	98	98	90	80	66	61
[EtOH]_0_ (g/L)	0	3.7	6.4	6.2	5.6	5.7	5.3	4.5	5.8	6.0
[HAc]_0_ (g/L)	10.7	13.9	15.5	16.9	17.7	17.7	17.3	18.9	17.9	17.1
[EtOH] (g/L)	14.2	15.9	15.2	15.9	15.5	15.5	15.3	16.7	13.7	14.7
Q_P_ (g/L/h)	1.2	1.3	1.4	1.3	1.2	1.1	1.0	0.7	0.4	0.3

Final cell viability and initial ethanol and acetic acid concentrations in repeated batch fermentations with encapsulated T18 in YPX 40 g/L with different acetic acid concentrations: A—0 g/L; B—4 g/L; C—8 g/L and D—10 g/L (35 °C, DO_0_ = 100, 50 g_drycells_/L, 150 rpm and pH 5.2). Values are triplicates averages, with less than 5% standard error. [EtOH]_0_ and [HAc]_0_ correspond to initial accumulated ethanol and acetic acid concentrations, respectively; [EtOH] is the final ethanol concentration and *Q*_P_ is ethanol productivity

In the fermentations with 4 g/L and 8 g/L acetic acid (Fig. 2b, c), encapsulated T18 completed 10 batches without reduction in ethanol yield and productivity. In fact, ethanol yield remained close to theoretical in both experiments, 0.46 and 0.45 g_ethanol_/g_xylose_, respectively. With a higher acetic acid concentration (10 g/L), it was possible to perform ten batches with total conversion of sugars (Fig. 2d). However, only the first six batches presented the same fermentation profile, with similar values of productivity. After the 7th batch of the set starting with 10 g/L of the inhibitor, cells became exposed to acetic acid concentrations of about 18 g/L, due to the accumulation of processed fluids that were retained in the gel beads when successive assays were run. Thus, at this point viability started to drop (Table 2), which caused an increase in fermentation time and, consequently, a decrease in ethanol productivity.

It is clear that higher acetic acid concentrations markedly reduced cell viability at the end of the process. On the other hand, since the acetic acid concentration in undetoxified hemicellulosic hydrolysates is generally around 6 g/L [4], yeast performance under these conditions was considered satisfactory.

### Repeated batch fermentations in a fixed-bed reactor using undetoxified sugarcane bagasse hemicellulose hydrolysate

Due to the good performance of encapsulated T18 in repeated batches with YPX medium in the presence of acetic acid, further studies with hemicellulose hydrolysate of sugarcane bagasse were carried out using the liquid fraction yielded after an acidic hydrolysis pretreatment of sugarcane bagasse. The sugarcane bagasse hemicellulosic hydrolysate was produced and kindly donated by Prof. Silvio S. Silva and Prof. Julio C. Santos research group (University of São Paulo, Brazil) and consisted of 9.7 g/L of glucose, 100.0 g/L of xylose, 8.3 g/L of arabinose, 6.5 g/L of acetic acid, 0.368 g/L of furfural and 0.016 g/L of hydroxymethylfurfural (HMF). Fermentations were run in a 100 mL fixed-bed jacketed bioreactor with and without medium recirculation using the hydrolysate supplemented with 10 g/L of yeast extract and 20 g/L peptone, 50 g_drycells_/L, pH 5.2 and 35 °C. Hydrolysate was not sterilized before the experiments. Control experiments were carried out with mini reactors, aiming to compare and validate the results with the fixed-bed bioreactor.

**Fig. 3:**
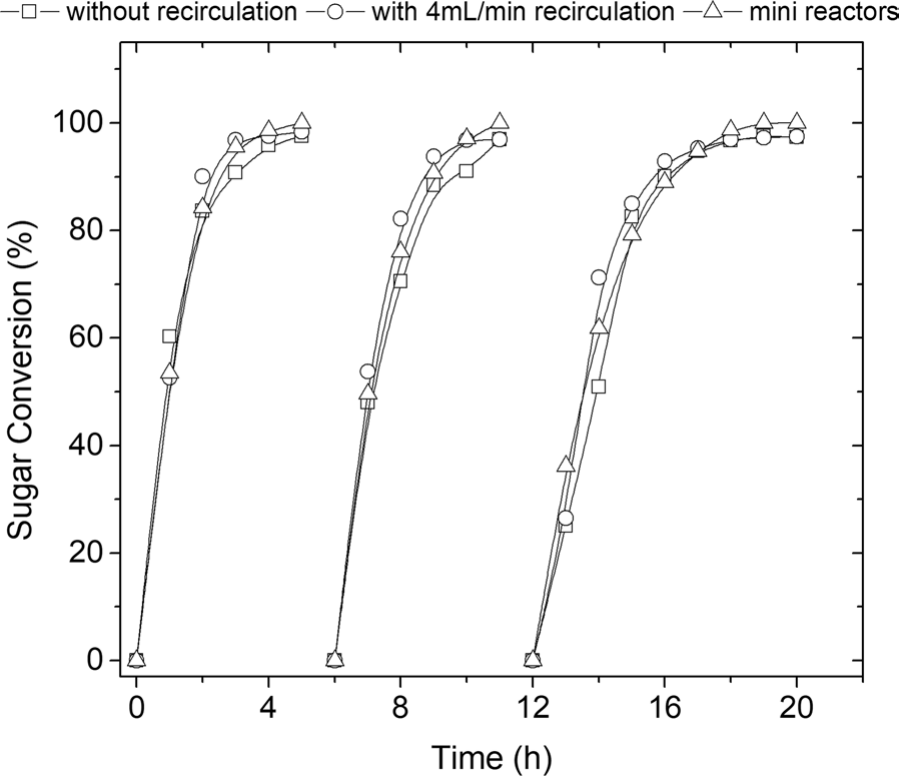
Repeated batch fermentations with encapsulated T18. Substrate: undetoxified sugarcane bagasse hemicellulose hydrolysate. Fixed-bed bioreactor (DO_0_ = 100; 50 g_drycells_/L, 35 °C, and pH 5.2), using mini reactors as control

T18 yeast was capable of fermenting all sugars in the hydrolysate, except arabinose. This pentose is only used to some extent by a few genetically modified *S. cerevisiae* strains [25], which is not the case for T18. After 4 h of fermentation in the first batch, and for all experimental conditions, all xylose and glucose were consumed, achieving high sugar conversion (Fig. 3), ethanol productivity and yield (Table 3), with negligible byproduct formation.

**Table 3:** Fermentation performance of encapsulated T18 using undetoxified hemicellulose hydrolysate in mini reactors and fixed-bed bioreactor

Batch	Recirculation			No recirculation			Mini reactors		
	1	2	3	1	2	3	1	2	3
*Y*_P/S_ (g/g)	0.38	0.32	0.35	0.34	0.32	0.35	0.37	0.31	0.38
*Q*_P_ (g/L/h)	5.7	3.9	2.5	4.6	3.5	2.9	5.0	4.3	3.3
*q*_P_ (mg_ethanol_/g_drycells_/h)	114	78	50	92	70	58	100	86	66
Final conversion (%)	98.3	96.9	97.4	97.5	96.9	97.3	95.3	95.9	95.0

Fermentation performance of encapsulated T18 using undetoxified hemicellulose hydrolysate in repeated batches in mini reactors and in fixed-bed bioreactor with and without medium recirculation (DO_0_ = 100, 50 g_cell_/L, 35 °C and pH 5.2). *Y*_P/S_ is gram of ethanol produced per gram of fermentable sugars consumed, Q_P_ is ethanol productivity and q_p_ is the specific ethanol productivity

In addition, no significant difference was observed between fermentations carried out in mini reactors and in the fixed-bed bioreactor. This suggests that, under fermentative conditions, the mini reactors reproduced the fixed-bed reactor results and were suitable for collecting reliable data at laboratory scale. Mini reactors allow the generation of a large amount of experimental data, using a simple monitoring procedure and preserving fermentative conditions. Fermentation studies can also be carried out in bioreactors or shake flasks, but they would demand larger volumes.

**Fig. 4:**
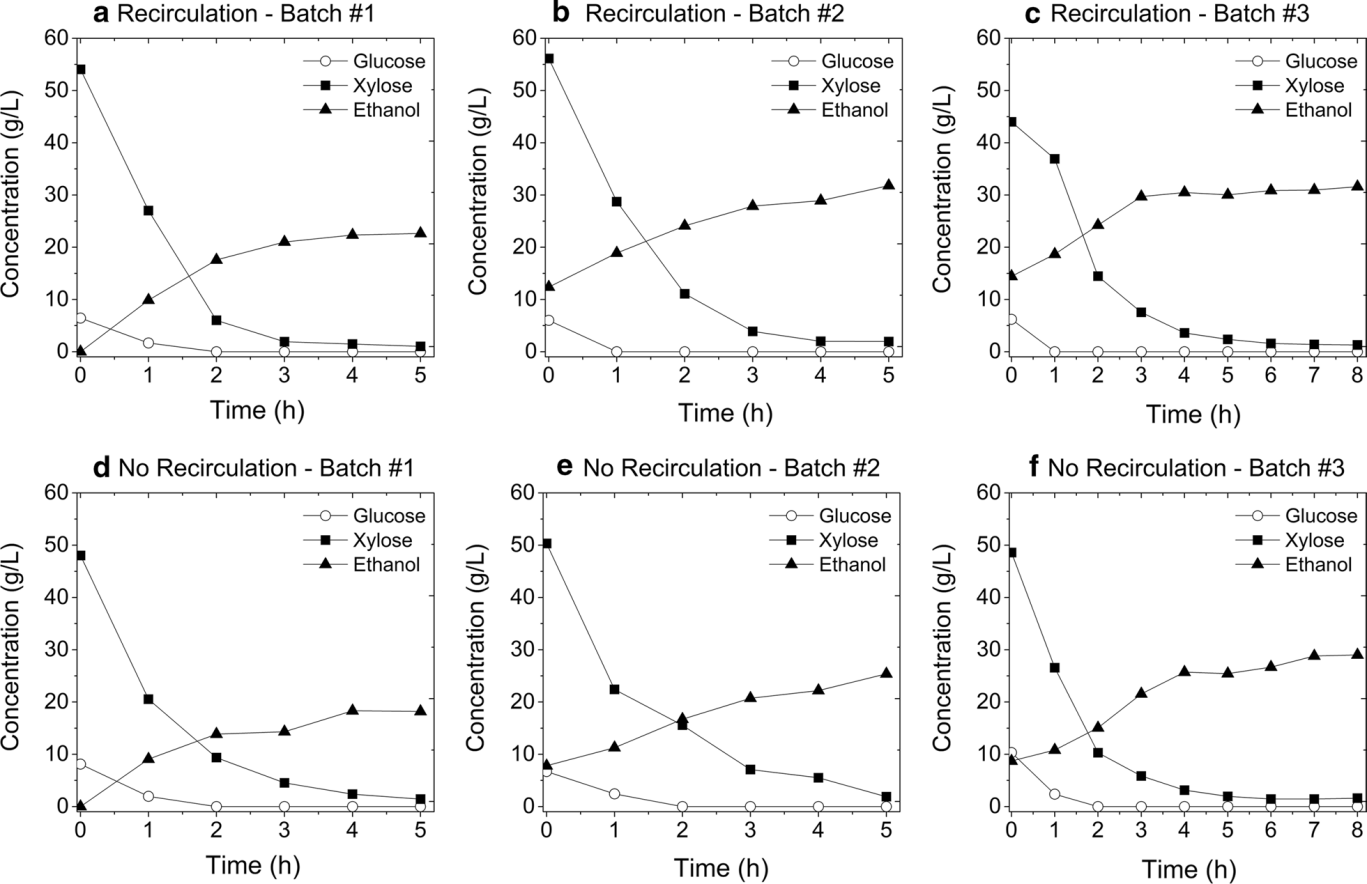
Profile of sugar consumption and ethanol production by immobilized T18 yeast during repeated batch fermentations of sugarcane bagasse hemicellulose hydrolysate in a fixed-bed reactor with and without medium recirculation. Conditions: DO_0_ = 100, 50 g_cell_/L, 35 °C, pH 5.2 and flow rate of 4 mL/min

Results in Fig. 4 and Table 3 show that medium recirculation during fermentation did not improve productivity for the fixed-bed reactor. The aim of medium recirculation was to increase the velocity of the fluid, thus reducing extra-particle mass transfer resistance. However, the same profiles were observed between fermentation without recirculation and with the application of a medium flow of 4 mL/min (residence time of 25 min), indicating that mass transfer through the external film was not the limiting step. It is important to point out that the fixed-bed reactor was operated at high cell loads, leading to an intense release of CO_2_, which, in turn, contributed to promote mixing of the fermentation medium. This could be one reason why no improvement was observed with recirculation. The application of higher flow rates, however, could result in leaching of cells from the gel beads.

## Discussion

In the search for yeasts that can ferment xylose efficiently some bottlenecks still have to be overcome: low xylose consumption rates, formation of byproducts such as glycerol and xylitol and low ethanol productivity. Taking this into account, T18 stands out as an excellent xylose-fermenting yeast, with a high fermentation rate and without the formation of byproducts.

Despite the good performance in xylose fermentation, it is evident that acetic acid strongly interferes with T18 metabolism. The negative influence of this inhibitor has been described in many reports in the literature [26]. Acetic acid pKa is 4.76; at lower pHs it is largely in non-dissociated form, diffuses through the membrane and strongly reduces intracellular pH, affecting cell metabolism [27]. At a pH higher than acetic acid’s pKa (in the case of the present work, pH 5.2), most of the acid molecules will be dissociated, and consequently less toxic to the yeast. Nevertheless, the results shown in Fig. 1a still indicate the need for further improvements of T18 tolerance to acetic acid.

The results obtained with immobilized cells, on the other hand, support the hypothesis that the alginate gel matrix protects the yeast cells from toxic inhibitors. This phenomenon has also been reported in the literature for immobilized native *S. cerevisiae* exposed to ethanol and acetic acid, but the mechanism of protection was not clear [11]. According to Lanza et al. [28], Ca-alginate gel has a negatively charged molecular network that restricts the entry of negatively charged compounds like acetic acid. The existence of a “sacrifice” outer cell layer acting as a barrier against stress factors, protecting the inner cell layers [29], and the intraparticle mass transfer limitations, causing the formation of a concentration gradient of the inhibitor, may also contribute to a certain extent to this protective effect.

Considering that the undetoxified hydrolysate was used in the fixed-bed bioreactor experiments, the results from the present work stand out even when compared to xylose fermentation in synthetic medium or in detoxified hydrolysates, which exhibited ethanol productivities below 0.8 g/L/h for recombinant *S. cerevisiae* strains [30, 31]. Encapsulated T18 productivities were between 2.5 and 5.7 g/L/h. The combined effects of protective shield provided by alginate membrane and operation at high load seemed to be effective to improve process performance using undetoxified hydrolysate-based fermentation medium.

However, ethanol yield in hydrolysate decreased, ~ 20% in comparison to the values achieved in YPX medium (Table 1). The presence of inhibitors in the hydrolysate and their synergic effects on cell metabolism probably contributed to the deviation of energy towards cell maintenance and stress defense [32, 33]. Similar behavior has been described by Zhang et al. [34] in the fermentation of sweet sorghum hydrolysate by recombinant *S. cerevisiae*, with a significant decrease in ethanol yield from YP medium (0.405 g/g) to hydrolysate (0.303 g/g).

The productivity also dropped significantly from the 1^st^ to the 3^rd^ batch using the hemicellulose hydrolysate and led to the early interruption of the sequence of batches. The lower number of batch repetitions achieved with hemicellulose hydrolysate (Table 3) when compared to the YPX medium (Table 1a), at the same cell concentration (50 g_drycells_/L), is probably due to the presence of other inhibitors in the hydrolysate, such as furfural and HMF. The levels of furfural and HMF in the hydrolysate (0.368 and 0.016 g/L, respectively) most likely affected the fermentation performance of the yeast negatively. Heer and Sauer [35] reported that concentrations of furfural above 5 mM (0.48 g/L) and of HMF above 20 mM (2.5 g/L) inhibit growth and metabolism of native *S. cerevisiae*. In addition, recombinant yeast strains with artificially engineered pathways could be more sensitive to this type of inhibitors, as previously observed for acetic acid [12]. It is also worth mentioning that the final ethanol concentration in the hydrolysate, due to its higher fermentable sugar content, reached about 30 g/L (Fig. 4), which is nearly the double of that achieved in the YPX medium. Ethanol is a well-known inhibitor of yeast metabolism and its critical inhibitory concentration depends on the strain and cultivation conditions, varying from 15 to 126 g/L [36].

The repeated batch fermentation process has several advantages, especially when the microorganism is inhibited by the product, such as in the case of ethanol. Cell recycling (with free and immobilized cells) in glucose-rich medium has been studied in literature [37, 38]. Repeated batch processes for 2G ethanol production with hemicellulose hydrolysate, on the other hand, have rarely been mentioned in the literature. Sanda et al. [39] studied recycling of genetically modified *S. cerevisiae* (free form) in rice straw hemicellulose hydrolysate (12 g/L glucose and 10 g/L xylose) that reached five fermentation cycles of 24 h before productivity started to decrease. However, the low sugar content led to a low ethanol titer. Antunes et al. [40], using the yeast *S. shehatae* UFMG-HM 52.2 immobilized in Ca-alginate gel, performed five repeated batches with similar performance in a fluidized bed reactor using detoxified sugarcane bagasse hemicellulose hydrolysate (31 g/L xylose, 0.3 g/L glucose and 1.6 g/L acetic acid). However, the yield and productivity dropped significantly from the sixth cycle. In addition, despite being a naturally pentose fermenting yeast, the fermentation cycle lasted 48 h, even with a high cell concentration in the reactor.

Thus, the main challenge for industrial feasibility of the application of immobilized cells and cell recycling in 2G ethanol production with hemicellulose hydrolysates is to increase the number of cell recycles in the repeated batch fermentations.

As shown in a previous study [41], the strategy of washing the encapsulated cells in between batches does not improve yeast performance. Analogously, the inclusion of detoxification treatments to reduce the concentration of inhibitory compounds has the drawback of increasing operation time and process costs. An alternative is to further improve the inhibitor tolerance of the yeast strain, e.g., by applying evolutionary adaptation or by introducing superior alleles conferring higher inhibitor tolerance identified by polygenic analysis.

## Conclusions

Benefits of yeast encapsulation in Ca-alginate gel go beyond its protective effect towards inhibitors. It allows to conduct fermentations in repeated batch mode with high cell density, exhibiting superior performance and fast xylose assimilation with both YPX medium and undetoxified hemicellulose hydrolysate (even for a worst-case scenario with respect to the production of inhibitors, using acid hydrolysis of sugarcane bagasse for biomass pretreatment). Together with the development of recombinant *S. cerevisiae* strains more tolerant to inhibitors, encapsulation proved to be a powerful tool to create a robust biocatalyst, able to ferment undetoxified lignocellulose hydrolysates and suitable for biocatalyst recycling. After validation at the pilot-scale, this technology could be scaled-up and used for industrial 2G ethanol production with repeated batch fermentations or in a continuous fermentation process.

## Methods

### Microorganism and inoculum

The recombinant yeast GSE16-T18SI.1 (T18) was used. T18 is a strain of *Saccharomyces cerevisiae* engineered for xylose consumption through the insertion of multiple copies of the *Clostridium phytofermentans* xylose isomerase gene originally in the background of the 1G industrial bioethanol strain Ethanol Red and subsequently developed further through genome shuffling with other genetic backgrounds [4, 42].

For all experiments, a pre-inoculum was prepared by adding a loop of stock culture in 3 mL of YPXD (yeast extract 10 g/L, peptone 20 g/L, xylose 10 g/L, glucose 10 g/L, pre-sterilized at 121 °C for 20 min) supplemented with 100 µg/mL of ampicillin. After incubation for 12 h (200 rpm and 30 °C), the pre-inoculum was poured into a 1-L flask containing 250 mL of YPXD (10 g/L of glucose and 10 g/L of xylose), supplemented with 100 µg/mL of ampicillin, and the inoculum was kept at 30 °C with shaking at 200 rpm for 24 h. Yeast cells were recovered by centrifugation (4500 rpm for 30 min) and used in either free or immobilized form in the fermentation runs.

### Yeast immobilization

T18 immobilization was performed by encapsulation in Ca-alginate gel according to the procedure described by Silva et al. [3]. Cal-alginate beads of small diameter (1-3 mm) were obtained by dropping a suspension containing 1% (w/w) of sodium alginate and 10% (w/w) of yeast (100% cell viability) into a coagulation solution (CaCl_2_ 0.25 M and MgCl_2_ 0.25 M) with a pneumatic extruder, adapted from Trovati et al. [43]. The procedure was performed aseptically in a biological safety cabinet. Alginate and coagulation solutions were pre-sterilized by autoclaving at 121 °C for 20 min. Due to the high content of water in the composition of the biocatalyst (85% moisture), after the addition of the beads the medium was diluted approximately 1.8 times. To avoid changes in pH and medium composition, the beads were cured for 12 h at 4 °C in a cure solution composed of fermentation medium without the carbon source.

### Batch and repeated batch experiments using mini reactors

Fermentation experiments were performed in flasks with 8 mL of reaction volume containing free or immobilized cells. The experiments with rich YPX medium were performed with 40 g xylose/L and pH 5.2, in the presence of different concentrations of acetic acid (0 to 11 g/L) using immobilized (OD_0_ = 100; 50 g_drycells_/L) or free cells (OD_0_ = 4 or 100; 2 and 50 g_drycells_/L, respectively). YPX medium was sterilized as previously described (“Microorganism and inoculum” section). Due to the dilution caused by addition of the beads, the medium was prepared with twice the final xylose and acetic acid concentrations, to ensure the desired initial concentrations in the fermentations. The experiments with sugarcane bagasse hemicellulose hydrolysate were carried out with immobilized cells (OD_0_ = 100; 50 g_drycells_/L), using crude hydrolysate without detoxification, prepared by acid hydrolysis with H_2_SO_4_ (100 mg/g_drybagasse_ and solid ratio of 10% at 121 °C for 20 min) [22] and kindly donated by Prof. Silvio S. Silva and Prof. Julio C. Santos (University of São Paulo, Brazil), supplemented with 10 g/L yeast extract, 20 g/L peptone and 100 µg/mL of ampicillin (to prevent contamination). Hydrolysate-based medium was not sterilized. The hydrolysate consisted of 9.7 g/L of glucose, 100.0 g/L of xylose, 8.3 g/L of arabinose, 6.5 g/L of acetic acid, 0.368 g/L of furfural and 0.016 g/L of hydroxymethylfurfural (HMF). Before use, pH was adjusted to 5.2 with Ca(OH)_2_, followed by filtration to remove suspended solids. All fermentations were carried out at 150 rpm, 35 °C and initial pH 5.2. Fermentations were monitored by measuring weight loss due to CO_2_ release as a function of time [4]. Cell recycling experiments were performed by addition of new fermentation medium after the removal of fermented medium at the end of each cycle. The end of a cycle was defined by the cessation of weight loss due to CO_2_ release.

### Repeated batch experiments in a fixed-bed bioreactor

**Fig. 5:**
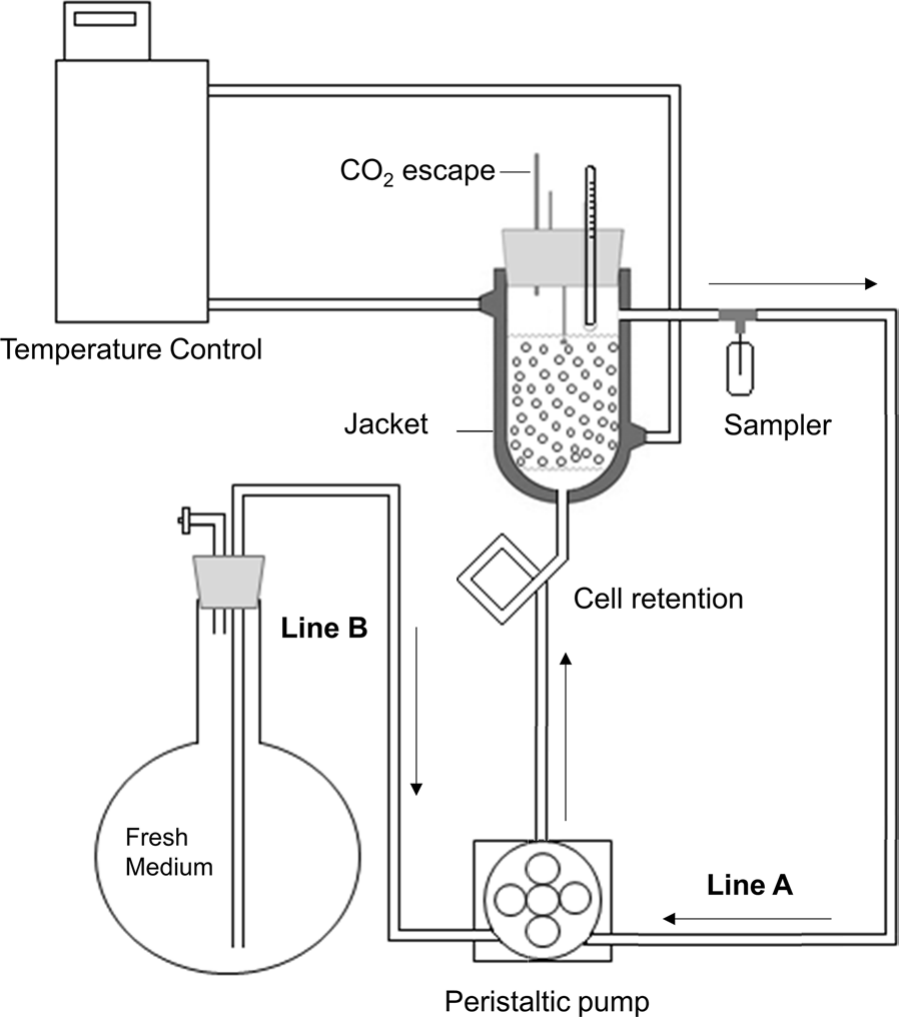
Scheme of fixed-bed reactor used for repeated batch fermentations with undetoxified xylose-rich hydrolysate using immobilized T18 yeast (*V* = 100 mL; 5 cm of diameter). Line A: operation with medium recirculation and fermentation broth removal. Line B: Addition of fresh medium

Bioreactor experiments were carried out in a fixed-bed jacketed reactor with 100 mL of reaction volume and 5 cm of diameter (Fig. 5), containing 50 g of beads (corresponding to 50 g/L of dry weight cells in the reactor) and 50 mL of undetoxified sugarcane bagasse hemicellulose hydrolysate (pH corrected to 5.2, as described in “Batch and repeated batch experiments using mini reactors” section), supplemented with peptone (20 g/L), yeast extract (10 g/L) and 100 µg/mL of ampicillin, presenting the same composition previously described (“Batch and repeated batch experiments using mini reactors” section).

The bioreactor loading was carried out in a laminar biological safety cabinet. The medium was fed with a peristaltic pump through the inferior part of the bioreactor and the experiment was carried out at 35 °C. Samples were taken to follow sugar and product content and, at the end of each batch, the fermented medium was recovered and fresh medium added.

To evaluate the occurrence of diffusional limitations, the fixed-bed reactor was operated with and without medium recirculation. Medium recirculation (4 mL/min) was provided by a peristaltic pump through Line A (Fig. 5). For medium charges, between batches, Line A was closed and medium was fed through Line B.

### Analytical methods

#### Substrate and product quantification

The concentrations of xylose, xylitol, glycerol, acetic acid, furfural, HMF and ethanol were determined by high-performance liquid chromatography (HPLC), using a Model 10AD (Shimadzu, Japan) chromatograph equipped with refractive index and UV–visible detectors. Analyses of xylose, ethanol, xylitol, glycerol and acetic acid were performed in an Aminex HPX87-H column, at 45 °C, with sulfuric acid (5 mM) in Milli-Q water as the eluent (0.6 mL/min). Hydrolysate samples were filtered with Sep-Pak^®^ C-18 (Waters) before injections. Furfural and hydroxymethylfurfural (HMF) were quantified using the C-18 (Beckman) column connected to a UV–visible detector (274 nm) using 0.8 mL/min acetonitrile/water 1:8 with 1% (v/v) acetic acid as eluent.

#### Cell concentration and viability

Cell concentration was determined by turbidimetry using a spectrophotometer (Ultrospec 2100 pro) at 600 nm and correlated with cell dry weight (g/L) through a calibration curve. Cell viability was evaluated by the methylene blue technique and counting in a Neubauer’s chamber. For the viability of immobilized cells, the alginate beads with the encapsulated cells were suspended in 8% (w/v) sodium citrate buffer (100 mg of beads per mL of buffer) under magnetic stirring and at room temperature to solubilize the gel beads. Cell viability was defined as the ratio between viable cells and total cells, counted in a defined space of the counting chamber.

### Calculations

Changes in substrate concentration (*C*_*S*_, in g/L) during the experiments in microreactors were estimated from the loss of CO_2_ mass ($\Delta m_{{{\text{CO}}_{ 2} }}$), according to Eq. 1:

1 $$C_{\text{S}} = C_{\text{Si}} - \left( {\Delta m_{{{\text{CO}}_{ 2} }} /Y_{{{\text{CO}}_{ 2} / {\text{S}}}} } \right),$$

where *C*_Si_ was the initial substrate concentration (in g/L) in YPX medium or hydrolysate at each batch. The value of 0.488 g of CO_2_ per g of consumed substrate was used as the theoretical yield coefficient $Y_{{{\text{CO}}_{ 2} /{\text{S}}}}$ based on the stoichiometric equation for ethanol production from sugars, assuming biomass formation as negligible [44]. In the experiments carried out in the bioreactor, *C*s was followed by sugar quantification in an HPLC. The performance indexes substrate conversion X (%), overall ethanol yield Y_P/S_ (g_ethanol_/g_substrate_), volumetric productivity *Q*_P_ (g/L/h) and specific volumetric productivity *q*_p_ (g/g_drycells_/h) were calculated according to Shuler and Kargi [45]. The specific productivity was estimated considering the initial mass of cells present corresponding to the total amount of beads added to the reactor. For repeated batch experiments, only the ethanol produced during a given batch was considered and the ethanol concentration from the previous batch, which remained inside the beads, was discounted from the final ethanol concentration for estimation of *Y*_P/S_, *Q*_P_ and *q*_P_.

## Supplementary information

**Additional file 1.** Process parameters for free and encapsulated T18 in YPX (yeast extract 10 g/L; peptone 20 g/L and xylose 40 g/L) in the presence of different acetic acid (HAc) concentrations (from 4 to 8 g/L) at 35 ºC, 150 rpm and pH 5.2.

**Additional file 2.** Performance of encapsulated T18 in YPX (xylose 40 g/L) in the presence of different acetic acid (HAc) concentrations (from 4 to 12 g/L) at 35 ºC, 150 rpm and pH 5.2.

## Data Availability

All data presented in this article, including Additional files 1 and 2, are also available from the corresponding author on reasonable request.

## Abbreviations

2G: Second generation
GMO: Genetically modified organisms
YPX: Yeast extract–peptone–xylose
YPD: Yeast extract–peptone–glucose
OD: Optical density
Y_P/S_: Ethanol yield (g/g)
Q_P_: Volumetric productivity (g/L/h)
q_P_: Specific volumetric productivity (g/g_drycells_/h)
HAc: Acetic acid
EtOH: Ethanol
HMF: Hydroxymethylfurfural
