# Supplementary material for: Repeated batches as a strategy for high 2G ethanol production from undetoxified hemicellulose hydrolysate using immobilized cells of recombinant Saccharomyces cerevisiae in a fixed-bed reactor
Source: Biotechnol Biofuels. 2020 May 11;13:85. doi: 10.1186/s13068-020-01722-y (PMC7216711; doi:10.1186/s13068-020-01722-y)
Supplement: Supplementary file 1 — Additional file 1. Process parameters for free and encapsulated T18 in YPX (yeast extract 10 g/L; peptone 20 g/L and xylose 40 g/L) in the presence of different acetic acid (HAc) concentrations (from 4 to 8 g/L) at 35 ºC, 150 rpm and pH 5.2. [file 13068_2020_1722_MOESM1_ESM.docx]

**Additional File 1**

**Repeated batches as a strategy for high 2G ethanol production from undetoxified hemicellulose hydrolysate using immobilized cells of recombinant *Saccharomyces cerevisiae* in a fixed-bed reactor**

Thais S. Milessi^a,b*^, Caroline L. Perez^c^, Teresa C. Zangirolami^a,c^, Felipe A. S. Corradini^c^, Juliana P. Sandri^c^, Maria R. Foulquié-Moreno^d,e^, Roberto C. Giordano^a,c^, Johan M. Thevelein^d,e^, Raquel L. C. Giordano^a,c*^

^a^ Department of Chemical Engineering, Federal University of São Carlos, Rodovia Washington Luís, km 235, 13565-905, São Carlos, SP, Brazil

^b^ Institute of Natural Resources, Federal University of Itajubá, Av. Benedito Pereira dos Santos, 1303, 37500-903, Itajubá, MG, Brazil

^c^ Graduate Program of Chemical Engineering, Federal University of São Carlos (PPGEQ-UFSCar), Rodovia Washington Luís, km 235, 13565-905, São Carlos, SP, Brazil

^d^ Laboratory of Molecular Cell Biology, Institute of Botany and Microbiology, KU Leuven, Kasteelpark Arenberg 31, B-3001 Leuven-Heverlee, Flanders, Belgium.

^e^ Center for Microbiology, VIB, Kasteelpark Arenberg 31, B-3001 Leuven-Heverlee, Flanders, Belgium.

*Correspondence should be addressed to Thais Milessi (thais.milessi@gmail.com)

Postal address: Institute of Natural Resources (IRN), Federal University of Itajubá

Av. Benedito Pereira dos Santos, 1303, 37500-903, Itajubá, MG, Brazil

**Table S1**: Process parameters for free and encapsulated T18 in YPX (yeast extract 10 g/L; peptone 20 g/L and xylose 40 g/L) in the presence of different acetic acid (HAc) concentrations (from 4 to 8 g/L) at 35ºC, 150 rpm and pH 5.2. All experiments performed in triplicate and the mean and standard error are shown

| T18 free cells | | | |  |
| --- | --- | --- | --- | --- |
|  | Ethanol (g/L) | Y_P/S_ (g/g) | Qp (g/L/h) |  |
| YPX 40 g/L | 16.3 ± 0.2 | 0.441 ± 0.006 | 1.36 |  |
| Acetic Acid 4 g/L | 16.2 ± 1.9 | 0.439 ± 0.054 | 0.68 |  |
| Acetic Acid 5 g/L | 18.3 ± 0.1 | 0.467 ± 0.003 | 0.38 |  |
| Acetic Acid 6 g/L | 16.5± 0.5 | 0.422 ± 0.018 | 0.23 |  |
| Acetic Acid 7 g/L | 16.3± 0.3 | 0.416 ± 0.005 | 0.17 |  |
| Acetic Acid 8 g/L | 15.9± 0.1 | 0.405 ± 0.025 | 0.13 |  |
| T18 encapsulated cells | | | |  |
|  | Ethanol (g/L) | Y_P/S_ (g/g) | Qp (g/L/h) |  |
| YPX 40 g/L | 14.4 ± 0.00 | 0.422 ± 0.066 | 3.61 |  |
| Acetic Acid 4 g/L | 15.4 ± 0.00 | 0.429 ± 0.000 | 1.93 |  |
| Acetic Acid 5 g/L | 16.2 ± 0.1 | 0.455 ± 0.009 | 1.35 |  |
| Acetic Acid 6 g/L | 15.5 ± 0.2 | 0.417 ± 0.004 | 1.30 |  |
| Acetic Acid 7 g/L | 14.3 ± 0.9 | 0.444 ± 0.024 | 1.19 |  |
| Acetic Acid 8 g/L | 15.6 ± 0.5 | 0.458 ± 0.019 | 1.30 |  |

Where Y_P/S_ is ethanol yield (g_ethanol_/g_xylose_) and Q_P_ is ethanol volumetric productivity (g_ethanol_/L/h)
