# Supplementary material for: Repeated batches as a strategy for high 2G ethanol production from undetoxified hemicellulose hydrolysate using immobilized cells of recombinant Saccharomyces cerevisiae in a fixed-bed reactor
Source: Biotechnol Biofuels. 2020 May 11;13:85. doi: 10.1186/s13068-020-01722-y (PMC7216711; doi:10.1186/s13068-020-01722-y)
Supplement: Supplementary file 2 — Additional file 2. Performance of encapsulated T18 in YPX (xylose 40 g/L) in the presence of different acetic acid (HAc) concentrations (from 4 to 12 g/L) at 35 ºC, 150 rpm and pH 5.2. [file 13068_2020_1722_MOESM2_ESM.docx]

**Additional File 2**

**Repeated batches as a strategy for high 2G ethanol production from undetoxified hemicellulose hydrolysate using immobilized cells of recombinant *Saccharomyces cerevisiae* in a fixed-bed reactor**

Thais S. Milessi^a,b*^, Caroline L. Perez^c^, Teresa C. Zangirolami^a,c^, Felipe A. S. Corradini^c^, Juliana P. Sandri^c^, Maria R. Foulquié-Moreno^d,e^, Roberto C. Giordano^a,c^, Johan M. Thevelein^d,e^, Raquel L. C. Giordano^a,c*^

^a^ Department of Chemical Engineering, Federal University of São Carlos, Rodovia Washington Luís, km 235, 13565-905, São Carlos, SP, Brazil

^b^ Institute of Natural Resources, Federal University of Itajubá, Av. Benedito Pereira dos Santos, 1303, 37500-903, Itajubá, MG, Brazil

^c^ Graduate Program of Chemical Engineering, Federal University of São Carlos (PPGEQ-UFSCar), Rodovia Washington Luís, km 235, 13565-905, São Carlos, SP, Brazil

^d^ Laboratory of Molecular Cell Biology, Institute of Botany and Microbiology, KU Leuven, Kasteelpark Arenberg 31, B-3001 Leuven-Heverlee, Flanders, Belgium.

^e^ Center for Microbiology, VIB, Kasteelpark Arenberg 31, B-3001 Leuven-Heverlee, Flanders, Belgium.

*Correspondence should be addressed to Thais Milessi (thais.milessi@gmail.com)

Postal address: Institute of Natural Resources (IRN), Federal University of Itajubá

Av. Benedito Pereira dos Santos, 1303, 37500-903, Itajubá, MG, Brazil

**Figure S1**: Performance of encapsulated T18 in YPX (yeast extract 10 g/L; peptone 20 g/L and xylose 40 g/L) in the presence of different acetic acid (HAc) concentrations (from 4 to 12 g/L) at 35ºC, 150 rpm and pH 5.2. All experiments performed in triplicate and the mean and standard error are shown. Immobilized Ethanol red yeast strain was used as negative control.
